# Spicy Herb Extracts as a Potential Improver of the Antioxidant Properties and Inhibitor of Enzymatic Browning and Endogenous Microbiota Growth in Stored Mung Bean Sprouts

**DOI:** 10.3390/antiox10030425

**Published:** 2021-03-10

**Authors:** Małgorzata Sikora, Urszula Złotek, Monika Kordowska-Wiater, Michał Świeca

**Affiliations:** 1 Department of Biochemistry and Food Chemistry, University of Life Sciences, Skromna Str. 8, 20-704 Lublin, Poland; malgorzata.sikora@up.lublin.pl (M.S.); urszula.zlotek@up.lublin.pl (U.Z.); 2 Department of Biotechnology, Microbiology and Human Nutrition, University of Life Sciences in Lublin, 20-704 Lublin, Poland; monika.kordowska-wiater@up.lublin.pl

**Keywords:** enzymatic browning, antioxidant capacity, microbiological purity, storage, herbs, consumer quality, infusion, phenolic compounds, bioaccessibility

## Abstract

The quality and shelf life of sprouts can be improved by postharvest application of water herb extracts. The effect of water infusions of marjoram, oregano, basil, and thyme on the phenolic content, antioxidant potential, and the microbiological and consumer quality of stored mung bean sprouts was studied. Compared to the control, the treatments increased total phenolic content. The highest amounts were determined in sprouts soaked in the thyme extract (6.8 mg/g d.m.). The infusions also inhibited the activity of enzymes utilizing phenolics, and marjoram and oregano were found to be the most effective. The increase in the level of phenolics was reflected in enhanced antioxidant properties (ability to quench cation radical ABTS^•+^, reducing and chelating power). Both total phenolics and flavonoids, as well as antioxidant capacities, were highly bioaccessible in vitro. All the natural extracts effectively reduced the growth of total mesophilic bacteria, coliforms, and molds (they were more effective than ascorbic and kojic acids). The treatments did not exert a negative influence on the sensory properties or nutritional value of the sprouts, and even improved starch and protein digestibility. These results are very promising and may suggest a wider used of natural extracts as preservatives of minimally processed food.

## 1. Introduction

Enzymatic browning of minimally processed “ready to eat” food such as mixed lettuce, sprouts, and peeled, washed, and shredded fruits and vegetables is a common and serious disorder not accepted by consumers. As a result, it generates enormous economic losses. For this reason, the food industry and scientists constantly look for tools to limit this undesirable phenomenon. For this purpose, chemical treatments are usually applied, e.g., with L-cysteine [1,2], citric acid, ascorbic acid [1], xanthone [3], lactic acid fermentation [4], sodium chloride, calcium chloride, sodium ascorbate [5], paeonol, and β-cyclodextrin [6]. Physical treatments include exposure to high-intensity light [7], various blanching methods [8], high-pressure processing [9], and a combination thereof, e.g., combination treatment of gamma irradiation and ascorbic acid [10] or mild heat shocks with chlorinated water with calcium ions or ascorbic acid [11]. Unfortunately, although they reduce the browning index, some of these methods have some disadvantages, for example, sulfites are potentially toxic and can therefore be considered a hazard to human health. Moreover, sulfite added as a preservative degrades thiamine (vitamin B_1_), which affects the nutritional quality of food [12]. Other disadvantages of the modern methods of food preservation, for example, high-pressure processing, are the high cost related to the initial capital investment, routine operating costs, maintenance, and subsequent amortization [13,14]. Blanching treatment has a negative impact on organoleptic properties (it involves loss of aroma and negatively influences the sensory properties associated with texture and color) [15]. Pulsed electric fields (PEFs) can cause damage to the structure (stress caused by PEFs when reaching the membrane potential can lead to loss of turgor and increase the possibility of extracting valuable components from cells) [16]. In turn, ionizing radiation can induce the generation of free radicals in addition to a certain loss of vitamins and proteins, which are especially sensitive to radiation [17].

A cheap, safe, and environmentally friendly strategy to prevent browning of minimally processed food is the postharvest application of natural compounds/extracts rich in bioactive compounds that are able to inhibit activation of enzymes responsible for this process (e.g., polyphenols, organic acids, sulfur compounds) [18,19]. This procedure not only extends the shelf life of minimally processed food, minimizing the generation of waste, but may also have a positive effect on its health-promoting properties. The use of natural extracts exhibiting antimicrobial properties can improve food safety and increase postharvest storability [20,21]. In addition, herbs can positively influence the sensory properties of food and tailor its pro-health properties, e.g., antioxidant, anti-inflammatory, and anticancer activities [21,22,23]. Finally, such an approach does not raise any objections in consumers who avoid products preserved with chemical compounds, for example, sulfites.

Herbs are characterized by intense taste, flavor, and color; hence, they are increasingly being used by various branches of the food industry [23]. They are a rich source of biologically active compounds, especially flavonoids and phenolic acids, which are able to maintain redox homeostasis, inter alia, by inhibition of the formation of reactive oxygen species with an unpaired electron and quenching those already produced. After consumption, they protect the human body against oxidative stress, i.e., an epidemiological factor in many diseases, especially cancer, cardiovascular, metabolic, autoimmune, and neurodegenerative diseases [24,25]. Due to these properties, herbs are widely used as natural preservatives, colorants, and additives for improving the pro-health potential of food [25].

The experimental hypothesis assumed that the activity of polyphenol oxidases and peroxidases during cold storage of mung bean sprouts could be effectively inhibited by the application of water extracts from the selected plant materials, which are a source of compounds inhibiting the activity of oxidoreductases. The most efficient inhibitors of enzymatic darkening and their optimal concentration were selected in a screening study. The effectiveness of functional solutions was tested during storage, and special emphasis was placed on the degree of enzymatic browning, microbiological quality, and potential positive effect on nutritional, antioxidant, and anti-inflammatory properties.

## 2. Materials and Methods

### 2.1. Chemicals

Citric acid, oxalic acid, lactic acid, ascorbic acid, kojic acid, MgCl_2_, CaCl_2_, ZnCl_2_, NaCl, KCl, catechol, guaiacol, hydrogen, peroxide, trichloroacetic acid, poly(vinylpyrrolidinone) (PVP), 2,2′-azino-bis(3-ethylbenzothiazoline-6-sulphonic acid (ABTS), AlCl_3_, NaOH, NaNO_2_, linoleic acid, ferrosine, FeCl_2_, FeCl_3_, K_3_[Fe(CN)_6_], α-amylase (52.7 U/mg), lipooxigenase (50 kU/mg), pancreatin (4 × UPS), pepsin (541 U/mg), bile extract, and Folin–Ciocalteau phenol reagent were purchased from Sigma-Aldrich (Poznan, Poland). Nutrient agar, MRS agar, yeast extract, chloramphenicol, VRBL, and other chemicals used for microbiological media were purchased from TL Ltd. (Łódź, Poland). All other chemicals were of analytical grade.

### 2.2. Sprouting Conditions

Seeds for sprouting mung beans were purchased from Legutko Breeding Company (Jutrosin, Poland). The seeds were disinfected in 1% *(v/v)* sodium hypochloride for 10 min, then drained and washed with distilled water until they reached neutral pH. After that, the seeds were placed in distilled water and soaked for 4 h at 25 °C. They were watered daily with 5 mL of Milli-Q water and germinated in the dark for 8 days in a growth chamber (23 °C, relative humidity 85%).

### 2.3. Screening Study

#### 2.3.1. Preparation of Functional Solutions

The materials tested belong to 3 groups: (i) pure chemicals (citric acid, oxalic acid, lactic acid, ascorbic acid, kojic acid), (ii) metal ions (MgCl_2_, CaCl_2_, ZnCl_2_, NaCl), and plant materials (oat bran from oat (*Avena sativa* L.), parsley (*Petroselinum crispum (Mill.) Fuss*), arugula (*Eruca vesicaria* L.), hibiscus (*Hibiscus* L.), common nettle (*Urtica dioica* L.), common chamomile (*Matricaria chamomilla* L.), white mulberry (*Morus alba* L.), green tea from Chinese tea leaves (*Camellia sinensis* L.), thyme (*Thymus vulgaris* L.), basil (*Ocimum basilicum* L.) oregano (*Origanum vulgare* L.), marjoram (*Origanum majorana* L)., St. John’s wort (*Hypericum perforatum* L.), bilberry (*Vaccinium myrtillus* L.), wheat bran from common wheat (*Triticum aestivum* L.), orange (*Citrus sinensis* L.), lemon (*Citrus limon* L.), and baby spinach (*Spinacia oleracea* L.). Chemically pure compounds and salts (sources of metal ions) were dissolved in water and analyzed at concentrations ranging from 8% to 0.001%. The plant material was ground using a laboratory grinder and 10 g samples were mixed with 90 mL of boiling distilled water (95 °C) and then allowed to cool. The infusions were tested at concentrations ranging from 10% to 0.0125%.

#### 2.3.2. Enzyme Extraction

All extraction procedures were conducted at 4 °C. For determination of polyphenol oxidase (PPO) and peroxidase (POD), 1 g of freeze-dried sprouts was ground with 10 mL of 100 mmol sodium phosphate buffer (pH 5.8 for PPO activity and 5.7 for POD activity—optimal pH in sprouts determined previously using sodium phosphate buffer) containing 0.2 g of PVP. The extracts were then homogenized, shaken for 30 min at 150 rpm., and centrifuged at 12,000× *g* at 4 °C for 30 min. The supernatants were used for further analysis [26].

#### 2.3.3. Enzyme Assay and Inhibition

Polyphenol oxidase activity was determined using catechol as a substrate [27]. The activity was expressed in units per g f.m., where U = 0.001 ΔOD420/min in the assay. Peroxidase activity was determined using guaiacol as a substrate [28]. The activity was expressed in units per g f.m., where U = 0.001 ΔOD470/min in the assay.

The inhibitory effects of the tested solutions on the activity of the enzymes were examined in the concentration range mentioned in Section 2.3.1. The corresponding control contained the same concentration of the enzyme with no inhibitor. The percentage inhibition was calculated using the following equation:

Inhibition (%) = (A_0_ − A*_i_*/ A_0_) × 100%,

where: A_0_: initial PPO activity (without the inhibitor); A*_i_*: PPO activity with the inhibitor.

The results were expressed in IC50 (extract concentration inhibiting 50% of enzyme activity).

### 2.4. Postharvest Treatments

Eight-day-old sprouts were manually collected and soaked for 2 h in the most effective inhibitor solutions prepared according to the procedure in Section 2.3.1 (M: extracts of marjoram leaves; O: extracts of oregano leaves; B: extracts of basil leaves; T: extracts of thyme leaves). The concentrations used in the study corresponded to 2 (2IC50) and 5 (5IC50) values of IC50 (half-maximal inhibitory concentrations) determined for POD (a key enzyme responsible for enzymatic browning in mung bean sprouts). The control samples were soaked in distilled water (C). Kojic and ascorbic acids were used as positive controls (K: solutions of kojic acid; AA: solutions of ascorbic acid). After soaking, the sprouts were kept in polypropylene boxes at 4 °C for 7 days. After storage, consumer evaluation and assays of the microbiological quality, enzyme activities, and browning index were performed immediately. The remaining sprouts were rapidly frozen, lyophilized, ground in a laboratory mill, sieved (60 mesh), and kept in polyethylene bags at −20 °C.

### 2.5. Browning Index

The browning index was determined using the procedure described by Ruangchakpet and Sajjaananta [29] with some modifications. Briefly, 1 g of fresh mung bean sprouts was homogenized in 15 mL of 10% trichloroacetic acid. After a 2 h incubation at 35 °C, the samples were centrifuged at 15,000× *g* at 25 °C for 15 min.

Changes in the color of the mung bean sprouts were measured using a High-Quality Colorimeter (Envisense). The values of L*, a*, b* were measured. The L* value indicates the lightness of the color, which ranges from 0 (dark) to 100 (white). A positive value of a* indicates a red color, while a negative value of a* indicates a green color. A positive value of b* indicates a yellow color, while a negative value of b* indicates a blue color.

The assessment of browning was carried out by calculating the following formula:

Browning index (BI) = [100 (w − 0.31)]/0.17,

where w = (a* + 1.75L*)/(5.645L* + a* − 0.3012b*).

### 2.6. Impact of Postharvest Treatment on Polyphenol Oxidase and Peroxidase

All extraction procedures were conducted at 4 °C according to the procedure described in Section 2.3.2. Polyphenol oxidase activity was determined using catechol as a substrate [27]. Peroxidase activity was determined using guaiacol as a substrate [28]. The results were presented as average fold changes compared to fresh samples.

### 2.7. Consumer Evaluation

The sensory properties of mung bean sprouts stored for 8 days were evaluated using a five-point method, where the worse sample received 1 point and the best sample received 5 points. The assessment included the following quality attributes: color, taste, aroma, and texture—where the weighting factor was 0.4, 0.2, 0.2, and 0.2, respectively. The evaluation was conducted by a group of 60 consumers (33 women and 27 men between the ages of 25 and 35). Coded samples were provided in polypropylene boxes to the panelists for evaluation at 20 °C.

### 2.8. Phenolic Compounds and Pro-Health Properties

#### 2.8.1. Extraction Procedures

For the extraction of phenolics, lyophilized samples of sprouts (500 mg of dry mass) were extracted for 1 h at room temperature (300 rpm) in a capped centrifuge tube with 5 mL of different solvents: 50% methanol, 60 mM HCl in 50% methanol, and finally with 60 mM HCl in 70% acetone. The mixture was centrifuged (15 min, 3000× *g*, 22 °C) and the supernatants from all steps were combined [30].

In vitro digestion was performed as described previously [31]. After digestion, the samples were mixed with pure methanol (1:1 ratio) and centrifuged (15 min, 6978× *g*). For undigested starch and protein assays, the pellets were additionally washed twice with ethanol.

#### 2.8.2. Low-Molecular-Weight Antioxidants

The amount of phenolics in samples obtained after chemical extraction and digestion in vitro was determined using the Folin–Ciocalteau reagent [32]. The amount of phenolics was expressed as mg gallic acid equivalents (GAE) per g of dry mass (d.m.).

Total flavonoid content was determined according to the method described by Haile and Kang [33]. A 1 mL sample of the test solution was mixed with 0.3 mL of NaNO_2_ (5%, *w/v*), and 0.5 mL of AlCl_3_ (2%, *w/v*) were added after 5 min. Flavonoid standard solutions of 100 μM were used. The sample was mixed and neutralized with 0.5 mL of 1 M NaOH solution 6 min later. The mixture was left for 20 min at room temperature. Thereafter, absorbance at 510 nm was measured. The total flavonoid content was calculated as a quercetin equivalent (QE) in mg/g of dry mass (d.m.).

#### 2.8.3. Antioxidant Properties

Antiradical properties were assayed according to Re et al. [34]. The free radical scavenging abilities were determined in samples obtained after chemical extraction and digestion in vitro and expressed as Trolox equivalents in mg per g of sprout dry mass (d.m.).

Reducing power was determined with the method developed by Pulido, Bravo, and Saura-Calixto [35] in samples obtained after chemical extraction and digestion in vitro and expressed as Trolox equivalents in mg per g of sprout dry mass (d.m.)

Chelating power (CHP) was determined using the method developed by Guo et al. [36]. The chelating properties were determined in samples obtained after chemical extraction and digestion in vitro and expressed as EDTA equivalents in µg EDTA per g of dry mass (d.m.).

#### 2.8.4. Anti-Inflammatory Properties

The lipoxygenase inhibitory (LOXI) assay was carried out using linoleic acid as a substrate with the method described by Szymanowska et al. [37] adapted to a microplate reader. One unit of LOX activity was defined as an increase in absorbance of 0.001 per minute at 234 nm (equivalent to the oxidation of 0.12 μmole of linoleic acid). The results were expressed in inhibitory units (UIs) per g d.w. One IU was defined as inhibition of 1U of enzyme activity in 1 min.

The ability of the extracts obtained after chemical extraction and digestion in vitro to inhibit cyclooxygenase-1 and cyclooxygenase-2 was determined using a COX colorimetric inhibitor screening assay kit (Cayman Chemical, No. 701050). The results were expressed in inhibitory units (UIs) per g d.w. One IU was defined as inhibition of 1U of enzyme activity in 1 min.

### 2.9. Nutritional Analysis

#### 2.9.1. Protein Content

Total protein was isolated from lyophilized samples according to Chen et al. [38]. The protein content in the extracts was determined with the Bradford method using bovine serum albumin as the standard protein [39]. The protein content was expressed in mg/g of dry mass (d.m.).

#### 2.9.2. Protein Digestibility In Vitro

The in vitro digestibility of protein (PD) was evaluated on the basis of protein content before (Pt) and after in vitro (Pr) [40]. Residues remaining after the digestion in vitro (Section 2.8.1) were extracted according to Chen et al. [38]. Protein digestibility in vitro was calculated as follows:

PD [%] = 100 − [(Pr/Pt) × 100]

where: PD = in vitro digestibility of the protein, Pt = total protein content, and Pr = content of proteins after in vitro digestion

### 2.10. Starch Analysis

#### 2.10.1. Starch Content

Total starch (TS) content was determined after hydrolysis of solubilized starch with thermostable α-amylase and amyloglucosidase (The Total Starch Kit, Megazyme) according to the AOAC Official Method 996.11. [41].

#### 2.10.2. Starch Digestibility In Vitro

The in vitro digestibility of starch was evaluated on the basis of total starch content (TS) and potentially resistant starch (RS) determined after digestion in vitro [40]. Starch contained in the residues remaining after the in vitro digestion was hydrolyzed with thermostable α-amylase and amyloglucosidase according to AOAC Official Method 996.11 (Resistant Starch Assay Kit, Megazyme) [41]. Starch digestibility in vitro was calculated as follows:

SD [%] = 100 − [(TS/RS) × 100]

where: SD = in vitro digestibility of starch, TS = total starch content, and RS = content of starch after in vitro digestion

### 2.11. Microbiological Quality

The following microbiological analyses were performed in accordance with Polish or European standards. For the tests, 10 g of mung bean sprouts were gently homogenized in a stomacher with 90 mL of Ringer’s solution for 10 min.

The total number of mesophilic bacteria was determined using the plate technique on nutrient agar according to PN EN ISO 4833-2 [42].

The number of yeasts and molds was determined with the plate technique on agar with glucose, yeast extract, and chloramphenicol according to PN-ISO 21527-1 [43]. Yeasts and molds were differentiated according to the morphology of colonies.

Coliforms were determined with the plate method on VRBL medium according to PN-ISO 4832 [44].

### 2.12. Statistical Analysis

All experimental results were the mean ± S.D. of three independent experiments (*n* = 9). One-way analysis of variance (ANOVA) and Tukey’s post hoc test were used to compare the groups (STATISTICA 13, StatSoft, Inc., Tulsa, OK, USA). Differences were considered significant at *p* < 0.05.

## 3. Results and Discussion

Enzymatic browning can reduce the organoleptic properties (such as color, smell, texture) and have a negative effect on functional (reduction of polyphenol compound content) and nutritional properties [45]. This process is commonly linked with polyphenol oxidase activities [46,47,48]. However, our research has shown that mainly peroxidase activity is involved in the generation of undesirable dark pigments in mung bean sprouts (Table 1). Similar results were also reported by Kim et al. [48] in a study describing the impact of phytoncide treatment on enzymatic browning in shredded lettuce (*Lactuca sativa* cv. Sacramento). The authors found that POD activity in fresh and 12-day-stored materials was 2.5 and over 3 times higher than PPO activity. Similarly, Ali, Khan, and Malik [49] showed that the POD activity in fresh lychee fruit was ca. 1.4-fold higher than PPO (this phenomenon was also observed after 7, 12, 21, and 28 days of storage).

The results of the effect of different potential inhibitors of enzymatic browning are presented in Table 1. Water extracts of marjoram, oregano, basil, and thyme were the most effective inhibitors (the lowest IC50 value) of peroxidase. Surprisingly, these extracts were approx. over 60 times more effective than citric acid, which is commonly used in the food industry to extend the postharvest shelf life by preventing oxidation processes causing negative color changes during storage of minimally processed food [1]. Among the studied solutions, the metal ions and infusions obtained from bilberry, citrus, and baby spinach did not inhibit POD activity. Similar results were obtained by Lin et al. [50], who studied the effects of different ions on polyphenol oxidase activity in potato. They observed that K^+^ was not an effective inhibitor of polyphenol oxidase. Mn^2+^ and Zn^2+^ activated the activity of PPO. A slight inhibition was observed only in the case of Ca^2+^ and Na^2+^. Arabaci [51] reported that ZnCl_2_ had no significant effect on enzyme activities in sorrel (*Rumex acetosa*). Gao, Liu, and Xiao [52] analyzed the effects of metal ions, surfactants, and common PPO inhibitors on the activity of purified polyphenol oxidase in common spider flower. They observed that Ba^2+^, Na^+^, and Zn^2+^ at both concentrations tested (1 and 5 mM) did not have any influence on PPO activity, whereas its activity was affected by other metal ions at the concentrations tested. Mn^2+^ at 1 and 5 mM as well as Mg^2+^ and Ca^2+^ at 1 mM enhanced the activity of PPO by 15–22%, but treatment with Ca^2+^ at a higher concentration resulted in loss of the activity by ca. 18%. The PPO activity was slightly inhibited (<15%) by Cu^2+^ and Fe^2+^, moderately inhibited (≤25%) by K^+^, Co^2+^, and Ni^2+^, and strongly inhibited (>30%) by Hg^2+^, Pb^2+^, and Ag^+^ at both concentrations.

**Figure 1:**
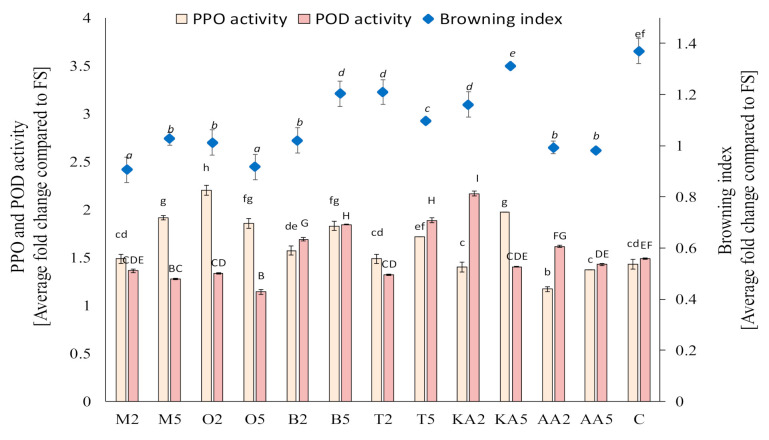
Effect of soaking in different solutions on the browning index and the polyphenol oxidase (PPO) and peroxidase (POD) activity in the control and treated mung bean sprouts after 8 days of storage. Means with different letters (lowercase: PPO activity, uppercase: POD activity, and italics: browning index) are significantly different (*n* = 9; α = 0.05). M2, M5: mung bean sprouts soaked in 2IC50 and 5IC50 water extracts of marjoram leaves, respectively; O2, O5: mung bean sprouts soaked in 2IC50 and 5IC50 water extracts of oregano leaves, respectively; B2, B5: mung bean sprouts soaked in 2IC50 and 5IC50 water extracts of basil leaves, respectively; T2, T5: mung bean sprouts soaked in 2IC50 and 5IC50 water extracts of thyme leaves, respectively; KA2, KA5: mung bean sprouts soaked in 2IC50 and 5IC50 solutions of kojic acid; AA2, AA5: mung bean sprouts soaked in 2IC50 and 5IC50 solutions of ascorbic acid; C: mung bean sprouts soaked in distilled water, FS: fresh sample.

Based on the screening study of the postharvest treatments of the sprouts in the storage experiment, marjoram, oregano, basil, and thyme infusions were selected. The other solutions also exerted a significant effect on PPO activity; however, these four materials were selected due to their potential activity against POD, which plays a key role of in enzymatic browning of mung bean sprouts. It was found that, after 7 days of storage, the activity of PPO was reduced only in samples treated with ascorbic acid; however, POD activity was significantly decreased in sprouts soaked in both oregano and marjoram infusions and the low-concentration thyme extract (Figure 1). Compared to the untreated sample, the application of the high-concentration oregano extract decreased POD activity by ca. 24%. The reduction of POD activity (an enzyme mainly responsible for enzymatic browning in mung bean sprouts) was reflected in the values of the browning index (Figure 1). The best results (comparable with those from the ascorbic acid treatment) were obtained for sprouts soaked with the marjoram and oregano infusions—there was no change in the color compared to the fresh samples. The highest degree of enzymatic browning was recorded in the untreated samples (an increase in BI by ca. 40% compared to fresh sprouts).

To date, studies of the limitation of undesirable effects caused by enzymatic browning have been mainly focused on pure chemical compounds or their mixture. For instance, ascorbic acid, L-cysteine, and sulfites [53] are usually effective; however, they are rarely applied to sprouted food. In our previous study, enzymatic browning was effectively limited in mung bean sprouts by ascorbic acid, which was able to decolorize (reduce) dark-pigmented quinones [26].

Postharvest application of natural extracts as antibrowning agents is limited; however, the treatment has recently become more popular, and some studies have confirmed its effectiveness. Sukhonthara et al. [54] evaluated an effect of rice bran extracts on the ability to inhibit enzymatic browning in potato and apple. The studied extracts effectively inhibited polyphenol oxidase (PPO) activity, which was reflected in reduced BI values after 6 days of storage; compared to untreated samples, a decrease by 40% and 23% in the apple and potato was recorded, respectively. Wheat bran extracts were also effective inhibitors of enzymatic browning in our previous study of shredded iceberg lettuce, where application of a 1% solution reduced BI by approx. 80% [55]. Wessels et al. [56] described the antibrowning effect of 36 plant extracts on minimally processed fresh apples. The extracts were applied to fresh-cut apple slices as 1% dipping solutions. Extracts obtained from horseradish root, grape seed, artichoke leaf, purslane, and oregano proved to be the most effective inhibitors of enzymatic browning; however, the results were significantly worse than those obtained for the reference compound (sodium bisulfite). A pistachio green hull extract was found to effectively inhibit mushroom tyrosinase, with IC50 values of 0.07%. The extract, which is a source of phloroglucinol, gallic acid, naringin, vanillic acid, catechin, and protocatechuic acid, applied at a concentration of 0.05%, exhibited properties comparable with those of ascorbic acid after 10 days of storage [57]. These results are comparable to those obtained after the application of the marjoram and oregano extracts in our study.

**Figure 2:**
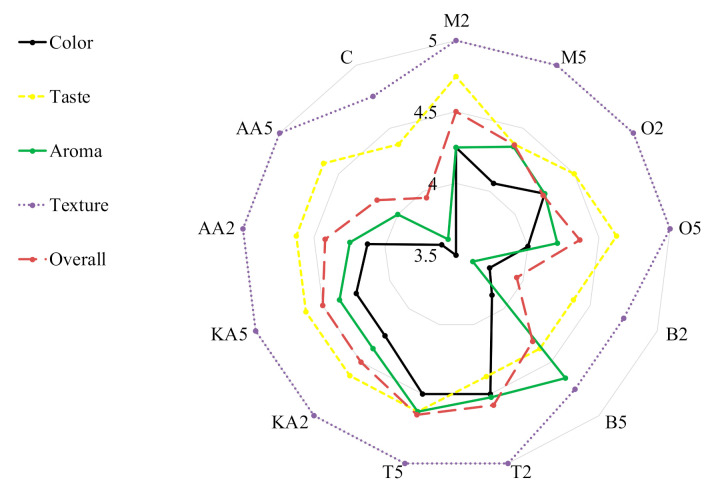
Consumer evaluation of analyzed stored mung bean sprouts. M2, M5: mung bean sprouts soaked in 2IC50 and 5IC50 water extracts of marjoram leaves, respectively; O2, O5: mung bean sprouts soaked in 2IC50 and 5IC50 water extracts of oregano leaves, respectively; B2, B5: mung bean sprouts soaked in 2IC50 and 5IC50 water extracts of basil leaves, respectively; T2, T5:mung bean sprouts soaked in 2IC50 and 5IC50 water extracts of thyme leaves, respectively; KA2, KA5: mung bean sprouts soaked in 2IC50 and 5IC50 solutions of kojic acid; AA2, AA5: mung bean sprouts soaked in 2IC50 and 5IC50 solutions of ascorbic acid; C: mung bean sprouts soaked in distilled water.

Antibrowning properties of natural extracts (pineapple juice, pineapple shell extract, and rice bran extract) were studied after application to banana slices and puree [58]. The best results were recorded for the bran extract—a 27% increase in brightness (L*) compared to untreated samples. The peroxidase activity and peroxidase gene expression were effectively inhibited in mung bean sprouts by the application of exogenous spermidine (180 μM). This treatment increased the content of total phenolics and the antioxidative activity of the sprouts [59]. Son, Moon, and Lee [19] confirmed the functionality of rhubarb juice for enzymatic browning reduction in apple slices; however, the effective concentrations were very high (up to 60%). In turn, Roldán et al. [60] showed that PPO activities in avocado fruit were significantly reduced by the application of different onion by-products, up to 58% in the case of 2% onion juice. A promising study confirming the inhibitory effects of clove essential oil (CEO) and eugenol (EUG) on browning and relevant enzymes of fresh-cut lettuce was performed by Chen et al. [61]. Postharvest treatment with 0.05% CEO and 0.05% EUG solutions suppressed PPO and POD activities and improved the quality of stored lettuce by inhibition of browning. Importantly, our results obtained for the sprouts treated with the marjoram and oregano extracts are comparable or even better that those reported in the cited study. Herbs not only can contribute to extending the shelf life of food but can also improve food sensory quality due to their intense aroma and color (Figure 2). Generally, the treatments did not influence the texture of the sprouts. The worst scores for color (3.5 and 3.75, respectively) and aroma (3.63 in both) were obtained in the control samples and those soaked in the low-concentration basil infusion. The highest overall quality was recorded for the sprouts soaked in both solutions of thyme (ca. 4.6); however, high results were also obtained in the samples treated with marjoram and kojic acid (Figure 2).

In the study conducted by Pace et al. [2], sensory analysis confirmed the effectiveness of L-cysteine treatment on fresh-cut lettuce. In the study, samples dipped in functional solutions obtained better scores for appearance, texture, and absence of browning and unpleasant odor than the control. These results are consistent with those obtained in our study, where all samples except the low-concentration basil-treated sprouts reached higher scores than the control. Contrarily, different antibrowning solutions (1% L-ascorbic acid, 0.5% L-cysteine at pH 7, 1% citric acid, and 0.5% 4-hexylresorcinol) had a negative effect on the quality of fresh-cut fennel. After 6 days of storage at 5 °C in air conditions, the applied antibrowning solutions (1% citric acid and 0.5% 4-hexylresorcinol) produced more severe browning than in the control; however, they did not negatively influence the aroma and crunchiness of the fresh-cut fennel.

**Figure 3:**
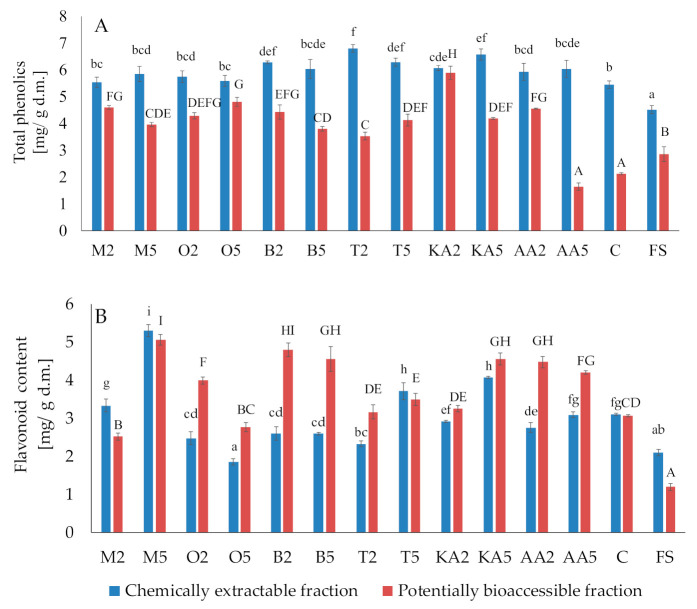
Effect of soaking in different solutions on total phenolic compounds **(A**) and flavonoids (**B**). Means with different letters (lowercase: in the chemically extractable fraction, uppercase: in the potentially bioaccessible fraction) are significantly different (*n* = 9; α = 0.05). M2, M5: mung bean sprouts soaked in 2IC50 and 5IC50 water extracts of marjoram leaves, respectively; O2, O5: mung bean sprouts soaked in 2IC50 and 5IC50 water extracts of oregano leaves, respectively; B2, B5: mung bean sprouts soaked in 2IC50 and 5IC50 water extracts of basil leaves, respectively; T2, T5: mung bean sprouts soaked in 2IC50 and 5IC50 water extracts of thyme leaves, respectively; KA2, KA5: mung bean sprouts soaked in 2IC50 and 5IC50 solutions of kojic acid; AA2, AA5: mung bean sprouts soaked in 2IC50 and 5IC50 solutions of ascorbic acid; C: mung bean sprouts soaked in distilled water, FS: fresh sample.

Generally, both treatments with the herb solutions caused an increase in the total phenolic content, compared to the control and fresh sprouts (Figure 3). The largest amounts of total phenolics were found in sprouts soaked in the thyme extract (6.8 mg/g d.m. and 6.29 mg/g d.m for the low- and high-concentration solution, respectively). Unfortunately, the phenolics were poorly accessible in vitro; however, the postharvest treatments causing a significant increase in their content (except ascorbic acid) compensated for these undesirable changes. The content of potentially bioaccessible phenolics in sprouts soaked in the marjoram and oregano extracts was ca. 2-fold higher than in the control samples. The highest content of flavonoids was recorded for the samples treated with the high-concentration marjoram solution (5.3 mg/g d.m. and 5.06 mg/g d.m. in the chemical and potentially accessible fraction, respectively). Contrary to phenolics, the flavonoids were well accessible in vitro. Compared to the control, their content in the basil-treated sprouts was higher by 56.86% and 49.02% in the low- and high-concentration variants, respectively (Figure 3).

Postharvest treatment with pistachio hull extracts (PHEs) and ascorbic–citric acids also increased phenolic content and antiradical properties in stored mushrooms. On day 10, samples treated with the pistachio green hull extracts and ascorbic–citric acids had higher total phenolic content by 32% and 22%, respectively [57]. A higher ability to quench radicals (by 11%) was recorded in samples treated with PHEs, while the treatment with ascorbic–citric acids caused a slight decrease in the activity (by ca. 3%). In our study, an increase in total phenolics was recorded in all samples (up to 25% in the case of the low-concentration thyme extract). Additionally, the increase in the antiradical properties was much more noticeable, i.e., up to 25% in the samples treated with the low-concentration basil extract (Table 2).

As reported in the literature, the applied solutions are a rich source of phenolic compounds [62]; thus, it was assumed that they may increase the pro-health properties of stored mung bean sprouts. It has been suggested that functional solutions are responsible for sprout fortification with phenolics; however, they rather act as elicitors, inducing de novo synthesis of phenolics. A 2.5-fold increase in the solution concentration was not reflected in a proportional increase in phenolics. Similar findings were reported by Świeca and Dziki [63] in wheat sprouts treated with *Salix daphnoides* bark infusions. A 5-fold induction in phenylalanine ammonia-lyase activity, reflected in a 3-fold increase in phenolics, was recorded in mung bean sprouts stored for 6 days at 4 °C [64].

Both the storage and the application of the functional solutions decreased the ability of the chemical extracts to inhibit COX2 activity (the worst results were found for the low-concentration marjoram solution) (Table 2). Inhibitors of COX2 are highly bioaccessible in vitro (an approximately 3-fold increase was recorded compared to the chemical extracts in the control): the activity in the samples of treated sprouts obtained after digestion was comparable with that recorded in the fresh sprouts. In contrast, an inverse relationship was observed in the case of the chemically extractable LOX inhibitors; the treatment significantly improved the ability to inhibit enzyme activity compared to the control. In the potentially bioaccessible fraction, an increase in the inhibitory activity was observed only in sprouts soaked in the water extracts of thyme, low-concentration ascorbic acid, and high-concentration kojic acid (Table 2). It was observed that the samples obtained after simulated gastrointestinal digestion were characterized by better antioxidant properties, e.g., the ability to quench the cation radical ABTS^•+^ and the chelation power (Table 2). It may be suggested that enzymes and chemical conditions occurring during digestion release flavonoids from the food matrix (Figure 3). A similar observation was recorded during digestion of soybean milk [65] or grapes [66]. Contrarily, the reducing power was significantly reduced by the digestion in vitro. It may be suggested that some components, e.g., ascorbic acid, may be disturbed during digestion, which was previously found in a study focused on storage of lettuce [30] and mung bean sprouts [26].

A similar behavior was recorded in 5-day-old mung bean sprouts stored for 7 days, where a ca. 53% decrease was observed [67]. The antiradical properties in chemically extractable fractions seem to depend on the total phenolic content. In turn, the increase in the antioxidant properties (especially chelating power) in the potentially bioaccessible fraction was significantly higher than the observed release of phenolics from the matrix. It may be suggested that the increase in the activity was caused by the release of bioactive peptides [68]. Previously, compared to phenolic-rich chemical extract, a significant increase in chelating power in potentially bioaccessible fractions was recorded in sprouted wheat [69] and stored lentil sprouts [70]. Additionally, Jakubczyk et al. [69] proved that peptides released from legumes possess strong chelating properties.

Generally, the effect of storage and postharvest treatments on the protein content was marginal, but some changes in its digestibility were clearly visible (Table 3). The effects of the postharvest treatments are obvious, because the lowest protein and starch digestibility values were recorded for the fresh and control stored sprouts. In our results, the protein content fluctuates around 180 mg/g. Comparable results were obtained by Swieca et al. [71], who reported that 4-day-old fresh mung bean sprouts contained 207 mg/g of protein. As in that study, our results confirm that the storage caused a slight decrease in total proteins, i.e., up to 5% in the samples treated with the low-concentration thyme extracts. Similarly, in the study conducted by Fattahifar et al. [57], postharvest treatments with aqueous pistachio (*Pistacia vera* L.) green hull extracts caused a slight reduction of total protein contents in mushrooms. Surprisingly, in our study, the treatment with the phenolic-rich extracts improved protein digestibility, e.g., the treatment with the low-concentration oregano extract increased protein digestibility up to 15%. It may be suggested that two mechanisms are employed. On the one hand, phenolics present in the functional solutions may interact with digestion enzymes and decrease nutrient digestibility [70,71]. On the other hand, they can limit the utilization of rapidly and easily digestible proteins and starch by inhibition of the growth of endogenous microbiota, which was significantly reduced by the postharvest treatments. It was observed that the storage also caused an increase in the starch content; however, it increased starch digestibility up to 13% (low-concentration marjoram extract). Improved digestibility of starch was observed in all treated samples, especially in the case of mung bean sprouts soaked in the water extract of marjoram leaves (13% increase in starch digestibility) (Table 3).

Herbs and spices are increasingly being used to extend the shelf life of unprocessed foods due to their antimicrobial properties; however, their essential oils are usually used [72,73,74,75,76]. Unfortunately, their application is limited by the very intense aroma, which is not always acceptable to consumers [77]. In our research, the infusions of the selected herbs, characterized by acceptable organoleptic properties, were applied. Additionally, such functional solutions are easy to prepare and, in addition to essential oils, contain other compounds exhibiting pro-health properties and reducing the undesirable endogenous microbiota [78,79].

The effect of the postharvest treatment on the microbiological quality of the stored mung bean sprouts is presented in Table 4. Importantly, the antimicrobial effect recorded in our study was very prominent. The total mesophilic bacterial count was reduced up to 78% by the application of the high-concentration basil extract. The plant water extracts decreased the content of Coliforms (except the high-concentration extract of thyme) and total mesophilic bacteria. Gutierrez et al. [80] reported that an oregano-containing solution (at a concentration of 250 ppm) significantly decreased total mesophilic bacterial count in stored carrots. A combination of oregano and thyme (125 ppm + 250 ppm) had the same effect as that observed for the oregano alone. These results suggest that it is possible to replace chlorine, which is commonly used in decontamination treatments, with natural essential oils. In the case of lettuce, a better effect was obtained using a mixture of essential oils (oregano and thyme at a concentration of 125 ppm + 250 ppm, respectively) than oregano (250 ppm) alone. Our study provided similar results: the water infusions of selected herbs were much more effective than commonly used chemicals, e.g., kojic acid or ascorbic acid. Moreover, ascorbic acid reduced total mesophilic bacteria by ca. 6%; nevertheless, it concurrently stimulated the growth of yeasts and molds, which significantly limits its use for preservation of minimally processed food. As reported by Booth and Stratford [81], the major problems in unheated foods are yeasts and molds, which can grow at pH values well below three. Generally, the natural extracts have proved to be more effective in the reduction of endogenous microorganisms than kojic acid and ascorbic acid, which are commonly used for extension of the shelf life of food. Statistically significantly higher mold and yeast counts were observed only in samples treated with the high-concentration marjoram infusion; however, the increase was still lower than that in samples treated with ascorbic acid.

## 4. Conclusions

The application of herb water infusion exerted a positive effect on the consumer quality of stored mung bean sprouts. The treated sprouts were characterized by higher content of phenolics, which was reflected in an increase in the antioxidant properties. The thyme extract was the most effective, and all the functional solutions were more effective than chemicals that are commonly used in the food industry (ascorbic and kojic acids). Importantly, the treatments limited the microbial growth and reduced enzymatic browning (inhibition of the activity of oxygenases) without any negative effect on the organoleptic quality (improved aroma). Additionally, functional solutions are cheap and may be produced from waste by-products (e.g., plant stems). The present results are very promising and may suggest a possibility of a wider use of natural extracts as preservatives of minimally processed food.

## Figures and Tables

**Table 1:** Effects of different ions, chemical compounds, and water extracts on peroxidase and polyphenol oxidase activity in mung bean sprouts.

		Peroxidase	Polyphenol Oxidase
Initial Activity (U/g f.m.)		13,750	325
Ions	MgCl_2_	Not effective	Not effective
IC50 (%)	CaCl_2_	Not effective	Not effective
	ZnCl_2_	Not effective	Not effective
	NaCl	Not effective	Not effective
	KCl	Not effective	Not effective
Chemical compounds	Citric acid	7.89	7.77
IC50 (%)	Ascorbic acid	0.05	0.062
	Kojic acid	0.009	0.008
	Oxalic acid	Not effective	Not effective
	Lactic acid	Not effective	7.69
Water extracts	Oat bran	8.14	5.68
IC50 (%)	Parsley	6.97	3.21
	Arugula	4.00	2.32
	Hibiscus	3.80	3.56
	Nettle	2.34	3.65
	Chamomile	1.28	Not effective
	White mulberry	1.05	4.21
	Green tea	0.32	4.85
	St. John’s wort	0.27	2.56
	Thyme	0.12	1.11
	Basil	0.11	1.23
	Oregano	0.09	1.36
	Marjoram	0.08	0.56
	Bilberry	Not effective	Not effective
	Wheat bran	Not effective	Not effective
	Orange juice	Not effective	Not effective
	Lemon juice	Not effective	Not effective
	Lemon pips	Not effective	5.62
	Orange pips	Not effective	5.36
	Baby spinach	Not effective	Not effective

**Table 2:** Anti-inflammatory and antioxidant properties of mung bean sprouts treated with antibrowning agents.

Sample		Cyclooxygenase 2 Inhibition (kUI/g)	Lipoxygenase Inhibition (kUI/g)	Antiradical Properties (mgTE/g)	Reducing Power (mgTE/g)	Chelation Power (mgEDTA/g)
**Chemically Extractable Fraction**	M2	6.1 ± 0.23 a	26.1 ± 2.78 b	4.62 ± 0.26 a	61.6 ± 0.40 cde	16.7 ± 0.41 bc
	M5	12.5 ± 0.45 b	62.6 ± 1.65 h	4.58 ± 0.18 a	62.3 ± 0.65 e	18.1 ± 0.44 de
	O2	15.8 ± 0.46 c	38.5 ± 1.25 ef	4.44 ± 0.25 a	62.8 ± 0.26 ef	17.5 ± 0.43 cd
	O5	17.8 ± 0.45 c	29.3 ± 0.51 bc	4.65 ± 0.19 a	61.5 ± 0.15 cde	17.0 ± 0.42 bc
	B2	21.1 ± 0.55 d	35.9 ± 2.98 de	5.45 ± 0.03 b	66.4 ± 1.15 h	16.8 ± 0.41 bc
	B5	16.0 ± 0.24 c	41.7 ± 3.12 f	4.67 ± 0.18 a	61.3 ± 0.38 cde	16.0 ± 0.41 bc
	T2	17.8 ± 0.50 c	50.8 ± 0.09 g	4.94 ± 0.25 ab	60.5 ± 1.62 cd	17.1 ± 0.42 bcd
	T5	11.6 ± 0.65 b	32.8 ± 2.21 cd	4.51 ± 0.30 a	64.4 ± 0.44 fg	18.9 ± 0.46 e
	KA2	17.4 ± 0.53 c	50.7 ± 0.04 g	4.51 ± 0.29 a	68.5 ± 0.40 i	17.6 ± 0.43 cd
	KA5	21.7 ± 0.01 d	32.0 ± 1.25 cd	4.47 ± 0.13 a	65.6 ± 0.42 gh	17.6 ± 0.43 cd
	AA2	23.9 ± 0.58 ef	35.6 ± 2.25 de	4.55 ± 0.14 a	62.1 ± 0.17 de	16.9 ± 0.41 bc
	AA5	18.2 ± 0.51 c	41.7 ± 2.25 f	4.95 ± 0.44 ab	60.3 ± 0.31 c	17.4 ± 0.43 cd
	C	21.6 ± 0.63 de	16.9 ± 0.62 a	4.44 ± 0.12 a	42.4 ± 0.33 b	13.5 ± 0.33 a
	FS	25.6 ± 0.52 f	51.1 ± 1.08 g	4.46 ± 0.10 a	40.5 ± 0.77 a	16.3 ± 0.40 b
**Potentially Bioaccessible Fraction**	M2	71.2 ± 0.34 a	51.2 ± 1.54 c	13.73 ± 0.09 a	25.8 ± 0.2 b	338 ± 8.27 i
	M5	71.2 ± 0.21 a	40.0 ± 0.54 b	13.15 ± 0.44 a	28.1 ± 0.33 d	219 ± 5.37 f
	O2	71.8 ± 0.34 a	39.2 ± 0.23 b	12.88 ± 0.64 a	27.8 ± 0.29 cd	196 ± 4.80 d
	O5	70.8 ± 0.34 a	53.9 ± 1.17 c	12.62 ± 0.44 a	27.1 ± 0.44 c	160.6 ± 3.93 b
	B2	71.8 ± 0.68 a	35.9 ± 4.85 b	13.26 ± 0.69 a	29.4 ± 0.66 f	216 ± 5.28 ef
	B5	71.0 ± 0.18 a	41.4 ± 0.10 b	13.38 ± 1.44 a	28.2 ± 0.37 de	425 ± 10.42 j
	T2	71.2 ± 0.52 a	73.7 ± 1.75 e	13.68 ± 0.47 a	27.9 ± 0.35 cd	134 ± 3.42 a
	T5	71.6 ± 0.36 a	69.4 ± 0.58 de	12.71 ± 0.39 a	29.1 ± 0.18 e	160 ± 3.92 b
	KA2	71.8 ± 0.34 a	48.8 ± 2.92 c	13.13 ± 1.28 a	27.9 ± 0.15 cd	295 ± 7.23 h
	KA5	71.4 ± 0.34 a	75.1 ± 1.51 e	13.00 ± 0.28 a	29.3 ± 0.50 f	202 ± 4.95 de
	AA2	71.0 ± 0.18 a	90.5 ± 2.50 f	12.76 ± 0.82 a	27.7 ± 0.40 cd	263 ± 6.44 g
	AA5	70.2 ± 0.68 a	36.7 ± 2.33 b	12.57 ± 0.23 a	27.0 ± 0.28 c	206 ± 5.06d ef
	C	71.2 ± 0.34 a	66.9 ± 1.32 d	12.90 ± 0.10 a	28.1 ± 0.19 d	162 ± 3.96 b
	FS	70.2 ± 0.34 a	19.9 ± 1.67a	12.43 ± 0.45 a	23.9 ± 0.40 a	179 ± 4.37 c

Means in the same column with different letters (within the extracts) are significantly different (*n* = 9; α = 0.05). M2, M5: mung bean sprouts soaked in 2IC50 and 5IC50 water extracts of marjoram leaves, respectively; O2, O5: mung bean sprouts soaked in 2IC50 and 5IC50 water extracts of oregano leaves, respectively; B2, B5: mung bean sprouts soaked in 2IC50 and 5IC50 water extracts of basil leaves, respectively; T2, T5: mung bean sprouts soaked in 2IC50 and 5IC50 water extracts of thyme leaves, respectively; KA2, KA5: mung bean sprouts soaked in 2IC50 and 5IC50 solutions of kojic acid; AA2, AA5: mung bean sprouts soaked in 2IC50 and 5IC50 solutions of ascorbic acid; C: mung bean sprouts soaked in distilled water; FS: fresh sample, TE: Trolox equivalent; EDTA: ethylenediaminetetraacetic acid equivalent.

**Table 3:** Nutritional properties of mung bean sprouts treated with antibrowning agents.

	Total Protein (mg/g)	Protein Digestibility (%)	Total Starch (mg/g)	Starch Digestibility (%)
M2	182 ± 6.59 ab	73.6 ± 1.17 efg	228 ± 5.69 bc	85.2 ± 1.74 d
M5	182 ± 6.79 ab	73.7 ± 0.48 efg	181 ± 9.13 a	85.6 ± 1.02 d
O2	186 ± 6.75 ab	76.2 ± 1.28 g	230 ± 12.81 bc	81.1 ± 2.78 bcd
O5	179 ± 6.81 ab	67.6 ± 4.49 abcd	238 ± 8.35 bcd	85.0 ± 4.05 d
B2	183 ± 6.92 ab	74.5 ± 2.88 fg	255 ± 3.70 cd	83.9 ± 2.91 d
B5	191 ± 7.08 b	70.9 ± 0.99 cdef	290 ± 11.15 f	75.7 ± 2.26 ab
T2	176 ± 6.38 a	72.7 ± 3.04 defg	232 ± 7.96 bc	80.1 ± 2.66 bcd
T5	184 ± 5.87 ab	73.3 ± 2.61 efg	247 ± 10.16 bcd	80.3 ± 1.91 bcd
KA2	183 ± 5.92 ab	72.5 ± 1.03 defg	262 ± 12.37 de	77.4 ± 2.98 bc
KA5	181 ± 6.38 ab	63.3 ± 1.34 a	285 ± 11.37 ef	83.6 ± 2.87 d
AA2	182 ± 6.24 ab	69.3 ± 1.26 bcdef	252 ± 20.32 bcd	82.5 ± 0.44 cd
AA5	180 ± 6.62ab	69.0 ± 2.14 bcde	225 ± 12.77 b	84.0 ± 0.86 d
C	185 ± 6.20 ab	64.8 ± 1.83 ab	231 ± 8.57 bc	75.5 ± 1.18 ab
FS	189 ± 5.56 ab	65.8 ± 0.97 abc	352 ± 3.70 g	71.5 ± 0.82 a

Means in the column with different letters are significantly different (*n* = 9; α = 0.05). M2, M5: mung bean sprouts soaked in 2IC50 and 5IC50 water extracts of marjoram leaves, respectively; O2, O5: mung bean sprouts soaked in 2IC50 and 5IC50 water extracts of oregano leaves, respectively; B2, B5: mung bean sprouts soaked in 2IC50 and 5IC50 water extracts of basil leaves, respectively; T2, T5: mung bean sprouts soaked in 2IC50 and 5IC50 water extracts of thyme leaves, respectively; KA2, KA5: mung bean sprouts soaked in 2IC50 and 5IC50 solutions of kojic acid; AA2, AA5: mung bean sprouts soaked in 2IC50 and 5IC50 solutions of ascorbic acid; C: mung bean sprouts soaked in distilled water; FS: fresh sample.

**Table 4:** Effect of postharvest treatment with functional solutions on the microbial quality of stored mung bean sprouts.

Count of Microorganisms (log10 CFU/g f.m.)			
Sample	Total Mesophilic Bacteria	*Coliforms*	Molds and Yeasts
M2	7.60 c	6.66 b	n.d.
M5	7.77 f	6.67 b	1.64 b
O2	7.56 bc	6.73 bc	n.d.
O5	7.94 g	6.83 cd	n.d.
B2	7.75 ef	6.89 de	n.d.
B5	7.48 b	6.86 cde	0.70 a
T2	7.70 de	6.95 ef	n.d
T5	8.09 h	7.16 h	1.11 a
KA2	8.29 j	7.63 j	0.48 a
KA5	8.33 k	7.79 k	0.30 a
AA2	8.12 i	7.03 fg	1.89 c
AA5	7.65 cd	7.51 i	2.83 d
C	8.13 i	7.07 g	n.d.

Means with different letters in the same column are significantly different (*n* = 9; a = 0.05). M2, M5: mung bean sprouts soaked in 2IC50 and 5IC50 water extracts of marjoram leaves, respectively; O2, O5: mung bean sprouts soaked in 2IC50 and 5IC50 water extracts of oregano leaves, respectively; B2, B5: mung bean sprouts soaked in 2IC50 and 5IC50 water extracts of basil leaves, respectively; T2, T5: mung bean sprouts soaked in 2IC50 and 5IC50 water extracts of thyme leaves, respectively; KA: mung bean sprouts soaked in kojic acid; AA: mung bean sprouts soaked in ascorbic acid; C: mung bean sprouts soaked in distilled water; WME: 5% water extract of marjoram leaves; WOE: 5% water extract of oregano leaves; WBE: water extract of basil leaves; WTE: water extract of thyme leaves; CFU: colony forming unit; n.d.: not detected; f.m.: fresh mass.

## Data Availability

Not applicable.
